# The Chemical Constituents and Anti‐Hyperlipidemia Effect of Salt‐Processed Fenugreek Seed

**DOI:** 10.1002/fsn3.70043

**Published:** 2025-02-19

**Authors:** Yang Qu, Yi Wang, Honghe Xiao, Mingyue Jiang, Qian Cai, Yi Liu, Yu Zheng, Baojie Zhang

**Affiliations:** ^1^ College of Pharmacy Liaoning University of Traditional Chinese Medicine Dalian China; ^2^ School of Medicine, Women's Hospital Zhejiang University (The Hosptal of Jilin) Changchun China

**Keywords:** gut microbiota, hyperlipidemia, salt processing, *Trigonella foenum‐graecum* L.

## Abstract

The seed of fenugreek (FS) was traditionally used in diets and as a spice in India, as well as medicine in China. It had anti‐diabetic and anti‐hypolipidemic effects. According to the theory of traditional Chinese medicine, the effects of FS were enhanced after salt processing. But the enhanced effect of salt‐processed fenugreek seed (SFS) on anti‐hyperlipidemia was not yet fully understood. By UPLC‐QTOF‐MS analysis, five flavonoids and six saponins were tentatively identified in SFS. Salt processing increased the dissolution of polysaccharides and trigonelline. FS and SFS significantly improved the serum biochemical indexes, including total cholesterol (TC), triglycerides (TG), and low‐density lipoprotein cholesterol (LDL‐C) of hyperlipidemic rats, promoted the excretion of TC and total bile acid (TBA), and downregulated aspartate aminotransferase (AST). According to the results of factor analysis, FS and SFS restored the severity of hyperlipidemia to a similar extent, and SFS enhanced the excretion of cholesterol more significantly. FS and SFS reduced the ratio of Firmicutes/Bacteroidetes (F/B), which was upregulated in HFD group. Additionally, SFS significantly increased the abundance of Ruminococcus_1, which was negatively correlated with blood lipid levels. Thus, to regulate gut microbiota and promote the excretion of cholesterol were the mechanisms of the effects of SFS on hyperlipidemia. The higher amounts of total polysaccharides and trigonelline in SFS than in FS led to their different effects.

## Graphical abstract

Salt‐processed increased the dissolution of polysaccharides and trigonelline from FS. FS and SFS had good therapeutic effects on HFD‐induced hyperlipidemia, and improved intestinal flora. SFS was more effective than FS in regulated cholesterol intake and metabolism.

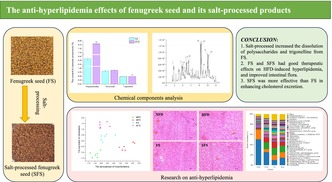

## 1 Introduction

Fenugreek seed (FS) is the seed of *Trigonella foenum‐graecum* L., Leguminosae sp. As traditional Chinese medicine (TCM), it has carminative, aphrodisiac, and tonic effects (Wu et al. 2020). In Chinese medicine theory, the processing of TCM could enhance the efficacy or reduce the toxicity. The salt processing is believed to lead the drug into the kidney and enhance the efficacy of tonifying liver and kidney. Salt‐processed FS (SFS) obtained from FS by infiltrating in salt‐water followed by stir‐frying has better carminative and aphrodisiac effects compared to FS, which is officially documented in the Chinese pharmacopeia.

Modern researches have shown that FS was rich in various active ingredients, including saponins, flavonoids, polysaccharides, alkaloids, and volatile oil, which contributed to its hypoglycemic, hypolipidemic, antioxidant, anti‐inflammatory, anti‐bacterial, and anti‐cancer effects, as well as cognitive improvements (He et al. 2021). Among these, the anti‐diabetic as well as anti‐hyperlipidemic activities of FS as well have been widely reported (Geberemeskel et al. 2019; Heshmat‐Ghahdarijani et al. 2020). Previous studies have found that FS could regulate serum levels of total cholesterol (TC), triglycerides (TG), low‐density lipoprotein cholesterol (LDL‐C), and high‐density lipoprotein cholesterol (HDL‐C) in patients with type 2 diabetes (Zahid et al. 2020). Moreover, FS modulated the abundance of Lachnospiraceae and Runinococcacea families in the intestinal microbes of mice, and alleviated hyperlipidemia by promoting fecal lipid excretion (Bruce‐Keller et al. 2020). However, SFS is more frequently sold in Chinese pharmacies. Early experimental research has demonstrated that salt‐processing changes the content of compounds in medicinal materials and enhances their hypoglycemic and hypolipidemic functions (Fan et al. 2021; Zhang, Fan, et al. 2022). However, the impact of salt‐processing on the anti‐diabetic and anti‐hyperlipidemic effects of FS has not been clearirfied yet. Therefore, our research group conducted a comparative analysis of the lipid‐lowering effects of methanol extracts of FS and SFS on hyperlipidemia mice. It showed that both methanol extracts of them had a regulatory effect on blood lipid levels, and the contents of TG and TC after administration of SFS were significant lower than those of FS. (Jiang, Ye, et al. 2020). However, it should be noted that the methanol extracts used in previous study do not represent all the active ingredients present in FS and SFS.

Thus, the powder of FS and SFS was applied to the efficacy assay on hyperlipidemia in this research and the main chemical constituents of them were qualitatively or quantitatively analyzed. Besides, the regulation effects on intestinal microbes of them, as well as correlation analysis between intestinal microbes and lipid metabolic parameters, were conducted to illustrate the underlying mechanism. The aim of this manuscript was to investigate whether the efficacy of FS on hyperlipidemia could be enhanced by salt processing and the mechanism underlying it.

## 2 Materials and Methods

### 2.1 Materials

FS was purchased from Mengcheng Boyaoqiancao Import & Export Co. Ltd., Anhui, China (batch no:2008132), and was identified by Professor Tianmin Wang as the seed of *Trigonella foenum‐graecum* L. In this study, SFS was prepared in laboratory according to the method in the literature (Jiang, Qu, et al. 2020). In detail, fenugreek was added to 0.05 g/mL NaCl solution (10:4 w/v), moistened for 4 h, and dried in an oven at 160°C for 10 min. High‐fat diet (HFD) was purchased from Shengmin Scientific Research Animal Farm, Nanjing, China. The fat emulsion was prepared by the laboratory. The nutritional composition for HFD and fat emulsion is shown in Table S1. The contents of serum and fecal lipid index and activity of transaminase were determined by kits produced by Nanjing Jiancheng Bioengineering Institute according to the manufacturer's instructions.

### 2.2 Analysis of Chemical Constituents

#### 2.2.1 Quantitative Determination of Total Flavonoids, Total Polysaccharides, and Trigonelline

To determine the content of total flavonoids, FS and SFS was accurately weighed, crushed with a pulverizer, and sieved with 65 mesh sieves. To this material, 75% ethanol was added at a ratio of 1:12 (w:v) and extracted at 75°C for 90 min for three times. Then, the content of total flavonoids were measured using sodium nitrite–aluminum nitrate method and rutin acted as standard substance to establish the standard curve according to the method reported in the literature (Cheng et al. 2021).

To determine the content of total polysaccharides, the residue after extraction of flavonoids was added distilled water at a ratio of 1:50 (w:v) and extracted at 95°C for 90 min for three times. The extracts were combined and concentrated to obtain the solutions of total saccharides of FS and SFS. Then the solution was added three times the volume of 95% ethanol. The supernatant was collected after rested overnight at 4°C, then dried with nitrogen to give the total oligosaccharides of FS and SFS. The total saccharides and total oligosaccharides were measured using phenol–sulfuric acid colorimetric method and glucose acted as standard substance to establish the standard curve (Zhang et al. 2019). The content of total polysaccharides in FS and SFS was calculated from the difference of the contents of total saccharides and total oligosaccharides.

To determine the content of trigonelline, the powder of FS and SFS was accurately weighed. To this material, petroleum ether was added at a ratio of 1:10 (w:v) and degreased by ultrasonic for 30 min for two times. The degreased powder was refluxed by 50% methanol at 75°C for 45 min for three times with ratios of 1:10, 1:20, and 1:20 (w:v), respectively. The sample solution was combined and concentrated in vacuum and reconstructed by 50% methanol. The content of trigonelline were determined on high performance of liquid chromatography (HPLC, Agilent 1100) according to the method described in our previous study (Jiang, Qu, et al. 2020). Briefly, HPLC separation was conducted on NH_2_ column (150 mm × 4.0 mm. 5 μm, Jiangshen Co. Ltd., Dalian, China) with mobile phase composed of 0.05% sodium dodecyl sulfonate in water, acetic acid and methanol with ratio of 80:0.1:20 at 1.0 mL/min. The detective wavelength was 265 nm. And the column temperature was 30°C. Three replicates were prepared per sample.

#### 2.2.2 Qualitative Analysis of Chemical Constituents

The UPLC analysis was performed used a Agilent Technologies 6540 UHD Accurate‐Mass Q‐TOF LC/MS (Agilent Technologies. Santa Clara, CA, USA), with a binary solvent delivery system, and an auto‐sampler. Chromatographic separation was performed on a C_18_ column (150 mm × 3.0 mm. 2.7 μm, YMC Co. Ltd.). The mobile phase was composed of 0.5% formic acid in water and acetonitrile with a gradient elution: 5% ~ 100% B, 0 ~ 15 min; 100% B, 15 ~ 20 min. The flow rate was 0.3 mL/min, and the column temperature was 40°C. The mass spectrometry was operated in positive ion mode used an ESI source. The operated parameters were shown in Table S2.

### 2.3 Animal Experiment

#### 2.3.1 Experimental Animals and Grouping

Twenty‐four male Sprague–Dawley (SD) rats (200 ~ 250 g) were purchased from Changsheng Biotechnology Co. Ltd., Liaoning, China. The rats were free to obtain food and water and were reared under environmentally controlled conditions (temperature 25°C ± 1°C and humidity 55% ± 5%) with a 12 h day/night cycle in the room. During the course of animal experimentation, the rats underwent regular weight assessments on a weekly basis, with subsequent computation of weight fluctuations to ascertain the extent of weight gain.

After 7 days of adaptation, 24 rats were randomly divided into four groups: NFD was offered normal diet, while HFD, FS, and SFS were offered HFD together with 2 mL fat emulsion per day for 6 weeks. In the last 2 weeks, FS and SFS were orally adminstrated powder suspension of FS and SFS (0.1 g/mL suspended in 0.5% CMC‐Na) at the dosage of 1 g/kg, respectively. NFD and HFD were given 0.5% CMC‐Na with the same volume.

#### 2.3.2 Sample Collection and Processing

After the 6‐week experiment, all rats were fasted overnight and anesthetized. All the rats suffered to ethyl carbamate at a dose of 1200 mg/g. Whole blood samples were immediately collected from the abdominal aorta, and centrifuged at 900 × g for 10 min to obtain the serum samples after placed at 25°C for 30 min. Fecal sample was collected and anhydrous ethanol was added at a ratio of 1:9 (w:v), ground with a tissue masher at 10,000 × g to make 10% tissue homogenate (Li et al. 2019). Moreover, cecal contents were collected and immediately frozen. All of the samples were stored at −80°C for subsequent analysis. The liver, testicles, thymus, and spleen were removed and weighed. The organ index was calculated as follows:

$$\text{Organ index}\;\left(\%\right)=\frac{\text{Weight of organ}}{\text{Weight of}\;\mathrm{rat}}\times 100\%$$

TC, TG, LDL‐C, HDL‐C, aspartate aminotransferase (AST), alanine aminotransferase (ALT), total bile acids (TBA), and total protein (TP) levels in serum or feces were assayed using corresponding assay kits (Nanjing Jiancheng Bioengineering Institute, Nanjing, China) according to the protocols described previously.

#### 2.3.3 Hematoxylin–Eosin (HE) Staining

The livers of rats were washed with saline and fixed with a 10% formalin solution for 24 h, dehydrated using gradient ethanol, cleared by xylene, embedded with paraffin, and sliced into 5 μm sections. Then dewaxed and incubated with hematoxylin for 30 min, stained with eosin solution for 1 min, dehydrated again, and sealed with neutral gum. Images were captured using a microscope (Olympus Corporation) at 200× magnification. Histological scores were measured using Kleiner's histological scoring system (Kleiner et al. 2005) by quantifying the size of adipocytes. Adipocyte size (μm^2^) was determined in three specimen selected for each group. Adipocyte size were measured using Image PRO (Media Cybernetics, MD, USA). This experiment was performed by Cairong Ming, Department of Pathology, Liaoning University of Traditional Chinese Medicine.

#### 2.3.4 Analysis of Short Chain Fatty Acids (SCFAs)

Cecal contents were weighed accurately. Then 50 μL of 0.2% H_3_PO_4_ solution contained 4‐methylvaleric acid as internal standard (0.668 mg/mL) was added. The samples were quickly sealed for analysis by DB‐WAX (DB‐1MS) capillary column (30 m × 0.25 mm, 0.25 μm, Agilent Corporation, USA) on Agilent 7890B‐5977B GC–MS instrument. The analysis conditions of GC–MS were described previously (Wang et al. 2023).

#### 2.3.5 16S rDNA Sequencing

PCR amplification of the bacterial 16S rDNA genes V3‐V4 region was performed using the forward primer 338F: 5′‐ACTCCTACGGGAGGCAGCA‐3′ and the reverse primer 806R: 5′‐GGACTACHVGGGTWTCTAAT‐3′. The 16S rDNA sequencing and analysis were performed as described previously (Wang et al. 2023).

### 2.4 Statistical Analysis

All data were presented as the mean value ± standard deviation (SD). Comparisons between two groups were conducted using a Student's *t*‐test to analyze the statistical significance of the differences. For analysis involving more than two groups, Tukey's test, Tamhane's T2 tests, or Mann–Whitney *U* tests were employed to assess the statistical significance of differences according to the results of homogeneity of variance and normality analysis. Values of *p* < 0.05 were considered to be statistically significant. And the factor analysis was used to differentiate the therapeutic effects of FS and SFS on HFD rats. The microbial diversity of samples was conducted with principal coordinate analysis (PCoA), Metastats test and linear discriminate analysis effect size (LEfSe) (Wang et al. 2023).

## 3 Results

### 3.1 The Analysis of Chemical Constituents in FS and SFS

#### 3.1.1 Contents of Main Chemical Constituents in FS and SFS

Table 1 shows that the contents of total flavonoids in FS and SFS were 0.84% and 0.88%, respectively, while the contents of total polysaccharides in FS and SFS were 5.37% and 8.06%, respectively. The content of polysaccharides increased significantly after salt processing (*p* < 0.01). The contents of trigonelline in FS and SFS were 0.48% and 0.49%, respectively, which showed a significant increase after salt processing (*p* < 0.01).

**TABLE 1:** Contents of chemical compositions in FS and SFS (%, mean ± SD, *n* = 3).

Chemical composition	FS	SFS
Total Flavonoids	0.84 ± 0.03	0.88 ± 0.02
Total Polysaccharides	5.37 ± 0.13	8.06 ± 0.02**
Trigonelline	0.48 ± 0.00	0.49 ± 0.00**

*Note:* Significantly different from FS.

** *p* < 0.01.

#### 3.1.2 Identification of the Components in FS and SFS

Besides the above three types of chemical components, steroidal saponins were another type of bioactive component related to the lipid‐lowering effect of FS (He et al. 2021). The content of steroidal saponins was 1% ~ 5.1% (Hadi and Mariod 2022) with various types, which underwent structural conversion during salt processing as reported in our previous research (Wang, Liu, et al. 2022). So a qualitative analysis based on UPLC‐QTOF/MS was applied to illustrate the different chemical compositions of FS and SFS.

**FIGURE 1:**
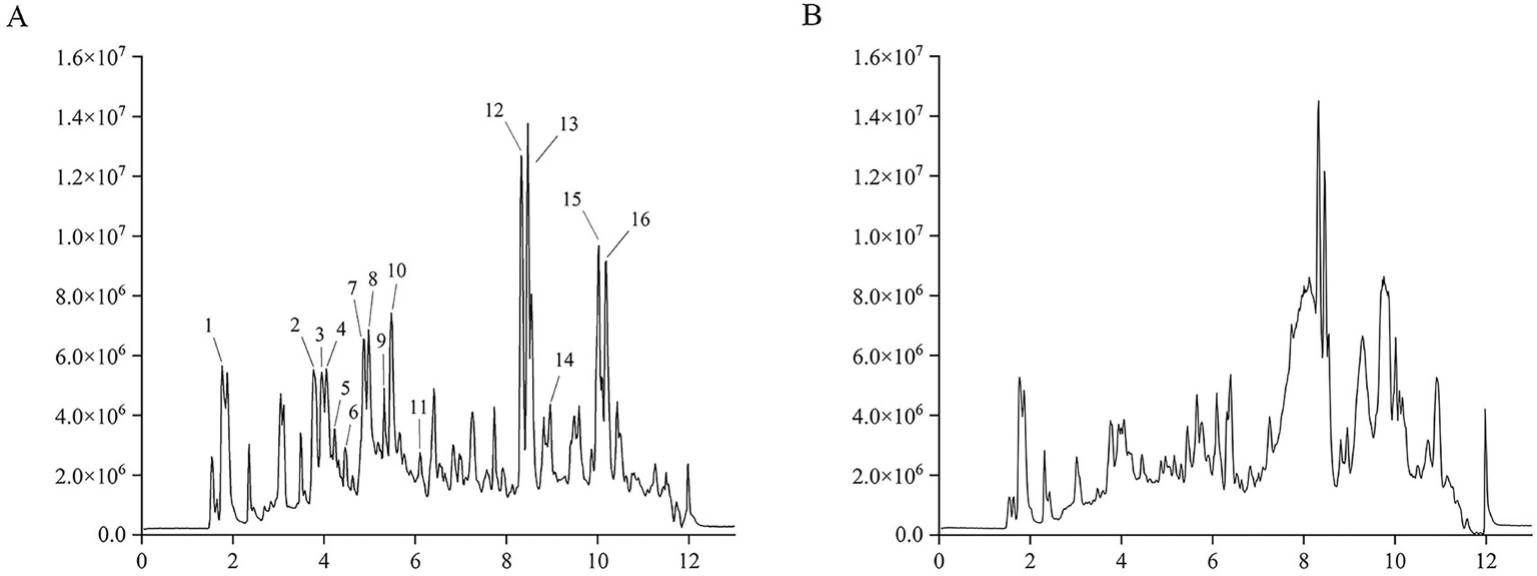
Total ion current (TIC) profiles of (A) FS and (B) SFS under positive mode (1, Trigonelline; 2, 5, Isomer of vicenin 2 (apigenin 6, 8‐di C‐hexoside); 3, 4, Apiin or Isomer of vicenin 3 (apigenin 8‐C‐xyloside‐6‐C‐glucoside); 6, Vitexin or Isovitexin; 7, Neogitogenin dihexosyl pentoside; 8, Gitogenon dihexosyl pentoside; 9, Tigogenin dihexosyl pentoside; 10, Diosgenin dihexosyl pentoside; 11, Tigogenin dihexosyl pentoside; 12, unidentified; 13, unidentified; 14, protoneogitogenin trihexosyl pentoside; 15, Vitamin E; 16, unidentified).

The TIC profiles of FS and SFS (Figure 1) show a significant difference in both peak numbers and response, which might also lead to the different efficacy of them. Thirteen compounds, including five flavonoids and six saponins, were inferred based on the accurate molecule mass and fragment ion, as well as data reported in the literature (Benayad et al. 2014; Guo et al. 2016; Khoja et al. 2022; Lang et al. 2010).

### 3.2 Effects of FS and SFS on HFD‐Fed Rats

#### 3.2.1 Effects of FS and SFS on Body Weight and Organ Index

**FIGURE 2:**
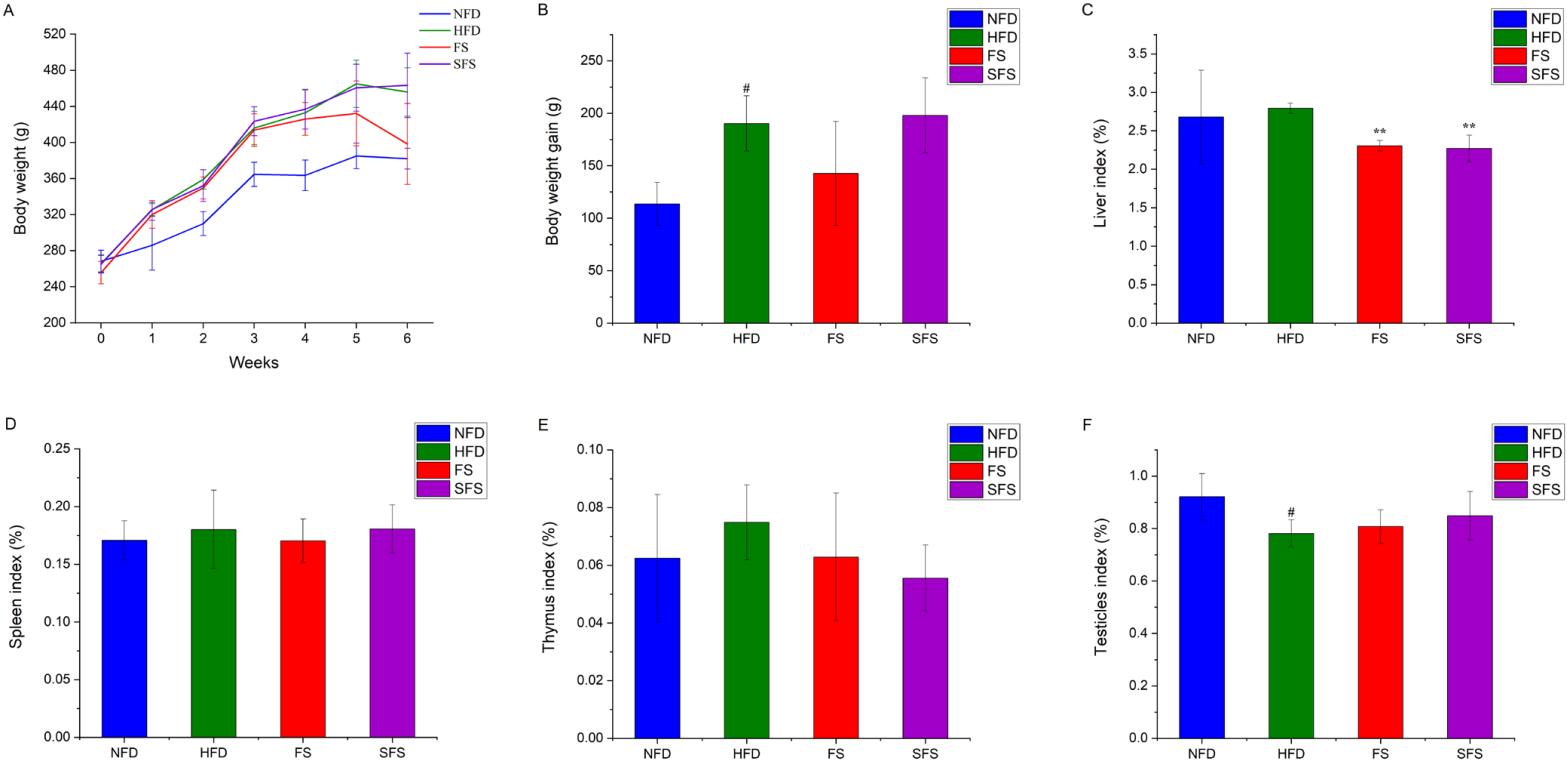
Effects of FS and SFS treatment on body‐weight and organ indexes in HFD‐fed rats. The (A) bodyweight, (B) body‐weight gain, (C) liver index, (D) spleen index, (E) thymus index, and (F) testicles index were shown, Values were expressed as mean ± SD in each group (*n* = 6). Significantly different from NFD group, ^#^ *p* < 0.05. Significantly different from HFD group, **p < 0.01.

As shown in Figure 2A, rats provided by HFD, including HFD, FS, and SFS groups, showed a significant growth in body weight compared with the NFD group (Figure 2B). As shown in Figure 2C–E, 6 week of HFD induced an increasing trend in the liver, spleen, and thymus indexes, though insignificantly, while FS and SFS led to the decrease of the liver index as compared with HFD. What is more, the HFD‐fed rats also presented a significant decrease in the index of the testicles compared with the NFD‐fed rats (*p* < 0.05; Figure 2F).

#### 3.2.2 Effects of FS and SFS on Serum Biochemical Indicators

**FIGURE 3:**
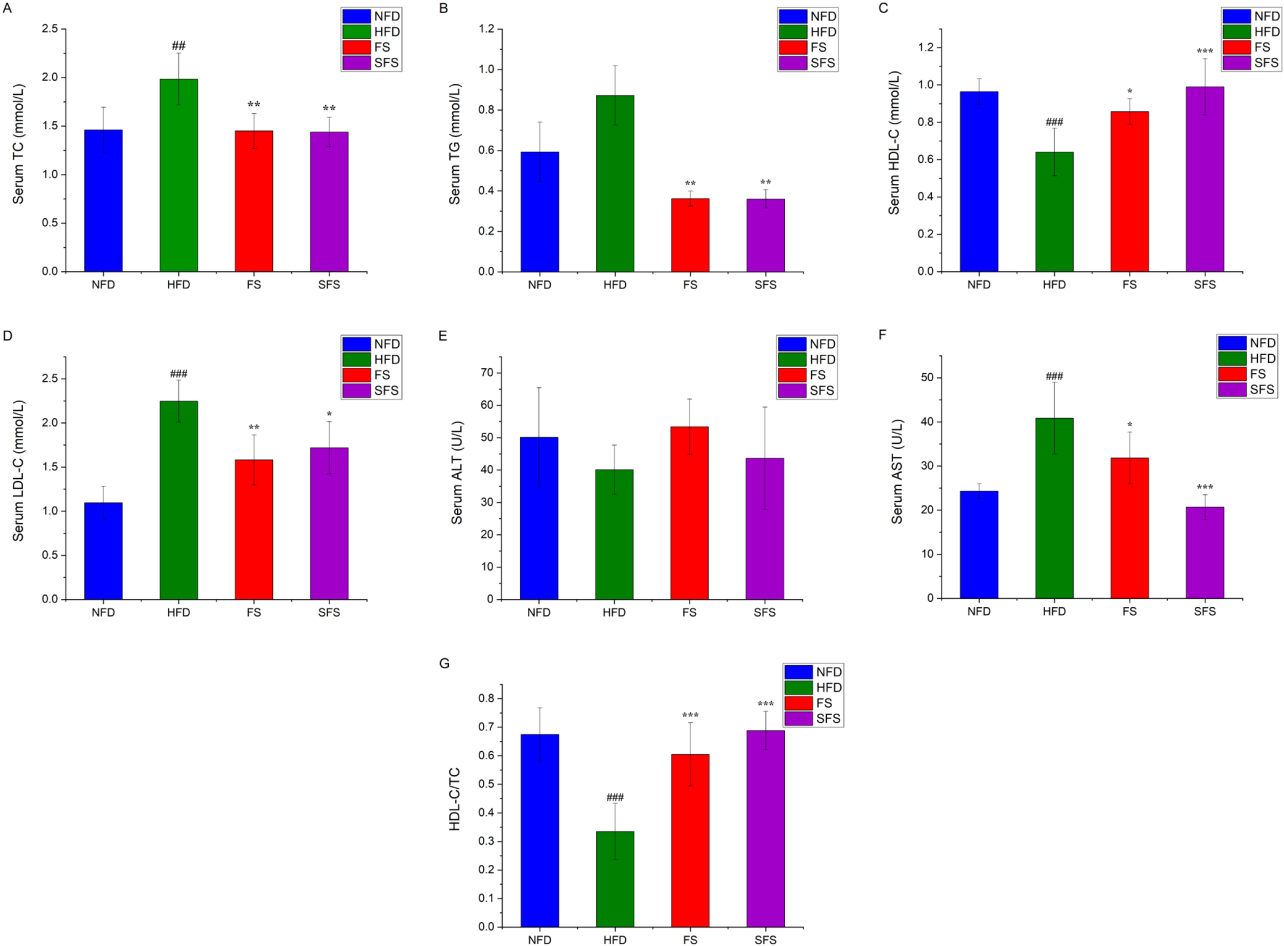
Effects of FS and SFS on serum biochemical indicators in HFD‐fed rats. The (A) TC, (B) TG, (C) HDL‐C, (D) LDL‐C, (E) ALT, (F) AST, and (G) HDL‐C/TC were shown. Values were expressed as mean ± SD in each group (*n* = 6). Significantly different from NFD group, ^##^ *p* < 0.01, ^###^ *p* < 0.001. Significantly different from HFD group, *p < 0.05, **p < 0.01, ***p < 0.001.

As shown in Figure 3, compared with the NFD group, the levels of TC, LDL‐C, and AST were significantly increased, and HDL‐C, HDL‐C/TC significantly decreased in the HFD group (*p* < 0.001). The FS and SFS interventions played similar roles in decreased TC, TG, LDL‐C, and AST levels in the HFD group of rats. Moreover, SFS was more pronounced relative to FS on the effect of restored serum HDL cholesterol levels. In addition, FS and SFS significantly increased HDL‐C/TC values in hyperlipidemia rats. (*p* < 0.001) (Figure 3).

#### 3.2.3 Effects of FS and SFS on Fecal Lipid and SCFAs

**FIGURE 4:**
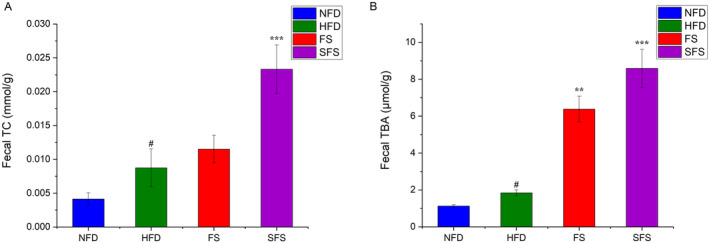
Effects of FS and SFS on the fecal lipid levels in HFD‐fed rats. The (A) TC and (B) TBA levels were shown. Values are expressed as mean ± SD in each group (*n* = 6). Significantly different from NFD group, ^#^ *p* < 0.05. Significantly different from HFD group, **p < 0.01, ***p < 0.001.

As shown in Figure 4, fecal TC and fecal TBA levels were significantly elevated in the HFD group of rats under the effect of HFD‐induced high triglyceride and high cholesterol intake. After treatment with FS or SFS, the fecal levels of TC and TBA were increased, which indicated that the lipid excretion was enhanced. Among them, SFS played a stronger role in promoting TG excretion. (*p* < 0.05).

**FIGURE 5:**
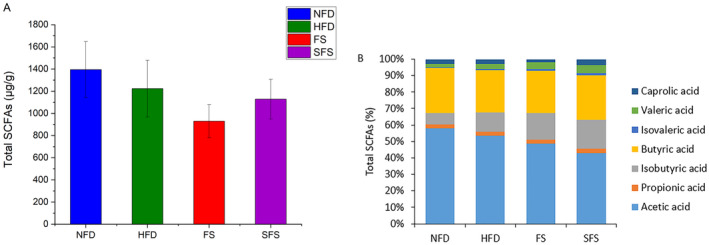
Effects of FS and SFS on the total contents of SCFAs in cecal contents in HFD rats. The (A) Total SCFAs levels and (B) Percentage of SCFAs were shown. Values are expressed as mean ± SD in each group (*n* = 6).

As shown in Figure 5 and Table 2, compared with the NFD group, HFD supplementation decreased the levels of total SCFAs, including branched‐chain fatty acids (BCFAs), although insignificantly. While FS and SFS had no significant effect on the levels of total SCFAs. SFS led to an increase in the abundance of isovaleric acid in the cecum of HFD rats (*p* < 0.05).

**TABLE 2:** The percentage of each kind of SCFAs and BCFAs of each groups (%, mean ± SD, *n* = 6).

Variety	NFD	HFD	FS	SFS
**SCFAs**				
Acetic acid	57.73 ± 3.51	55.34 ± 15.50	49.93 ± 8.43	42.90 ± 4.22
Propionic acid	2.40 ± 0.34	2.65 ± 0.20	2.38 ± 0.33	2.50 ± 0.25
Butyric acid	27.80 ± 2.72	24.36 ± 8.86	25.21 ± 5.15	26.56 ± 2.45
Valeric acid	2.16 ± 0.20	3.10 ± 1.23	3.80 ± 1.12	4.89 ± 0.84
Caproic acid	2.76 ± 0.54	2.58 ± 2.20	1.84 ± 1.29	3.52 ± 1.52
**BCFAs**				
Isobutyric acid	6.78 ± 1.12	11.21 ± 4.08	15.65 ± 5.95	18.10 ± 4.12
Isovaleric acid	0.37 ± 0.11	0.73 ± 0.34	1.19 ± 0.47	1.53 ± 0.48*

*Note:* Significantly different from HFD group.

* *p* < 0.05.

### 3.3 Factor Analysis Based on Biochemical Indicators as Variables

**FIGURE 6:**
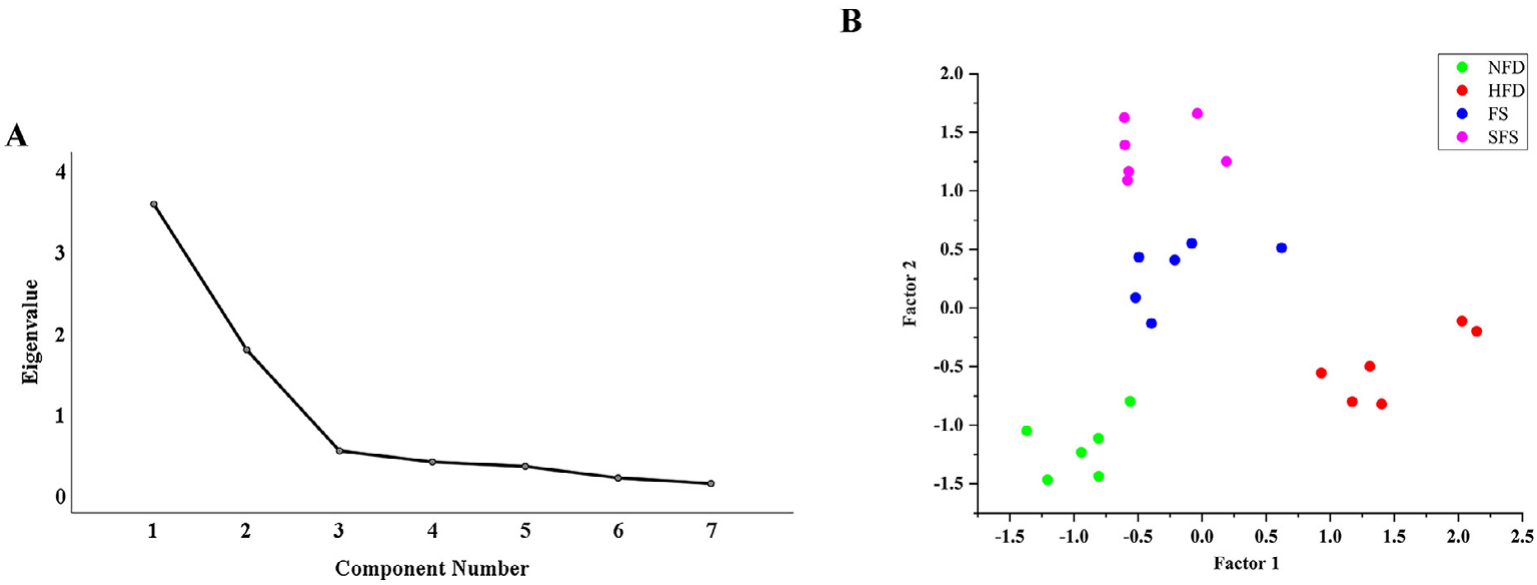
Factor analysis of biochemical indicators (A) Scree plot depicting the eigenvalues of the factors extracted by factor analysis used biochemical indices as variables. (B) Scatter plot of the factor analysis.

Since the difference of FS and SFS was not significant based on ANOVA and pos‐hoc analysis, factor analysis was performed with the levels of TC, TG, HDL‐C, LDL‐C, and AST in serum, as well as TC and TBA in feces as variables to analyze the different efficacy of FS and SFS. Bartlett's test of sphericity confirmed a correlation between the variables (*p* < 0.01), and the KMO measure of sampling adequacy was 0.771 (Table S3), indicating that these variables were suitable for factor analysis. A scree plot was used to determine the number of factors to extract; factors that had an eigenvalue greater than 1.0 were retained (Table S4). In this case, two factors were extracted, accounting for 76.625% of the total variability in the dataset (Figure 6A).

Variables with loading above 0.5 or below −0.5, which had a high influence, were selected for interpretation. The results of the factor analysis were summarized in Table 3. Factor 1 was labeled as “The severity of hyperlipidemia” and was characterized by serum TC, TG, HDL‐C, LDL‐C, and AST, all of which are associated with the development of hyperlipidemia. Factor 2 was largely dependent on fecal TC and TBA, indicating the excretion of cholesterol.

**TABLE 3:** Rotated component matrix of factor 1 and factor 2 based on maximum variance method.

	Factor 1	Factor 2
Serum TC	0.787	−0.199
Serum TG	0.603	−0.599
HDL‐C	−0.772	0.253
LDL‐C	0.855	0.318
AST	0.800	−0.250
Fecal TBA	−0.201	0.932
Fecal TC	−0.014	0.938

The distribution pattern of different study groups on the two factors was visualized in a plot (Figure 6B). Each group clustered well, suggested less differences within groups. The spots for the HFD group were far from the coordinate origin on factor 1, indicating that HFD greatly promoted the development of hyperlipidemia, while FS and SFS restored this effect comparably. On factor 2, the spots for the SFS group were farther from the coordinate origin than those for the FS group, indicating that the enhanced effect of excretion of cholesterol by SFS was stronger than that of FS.

### 3.4 Histopathological Analysis of Liver

**FIGURE 7:**
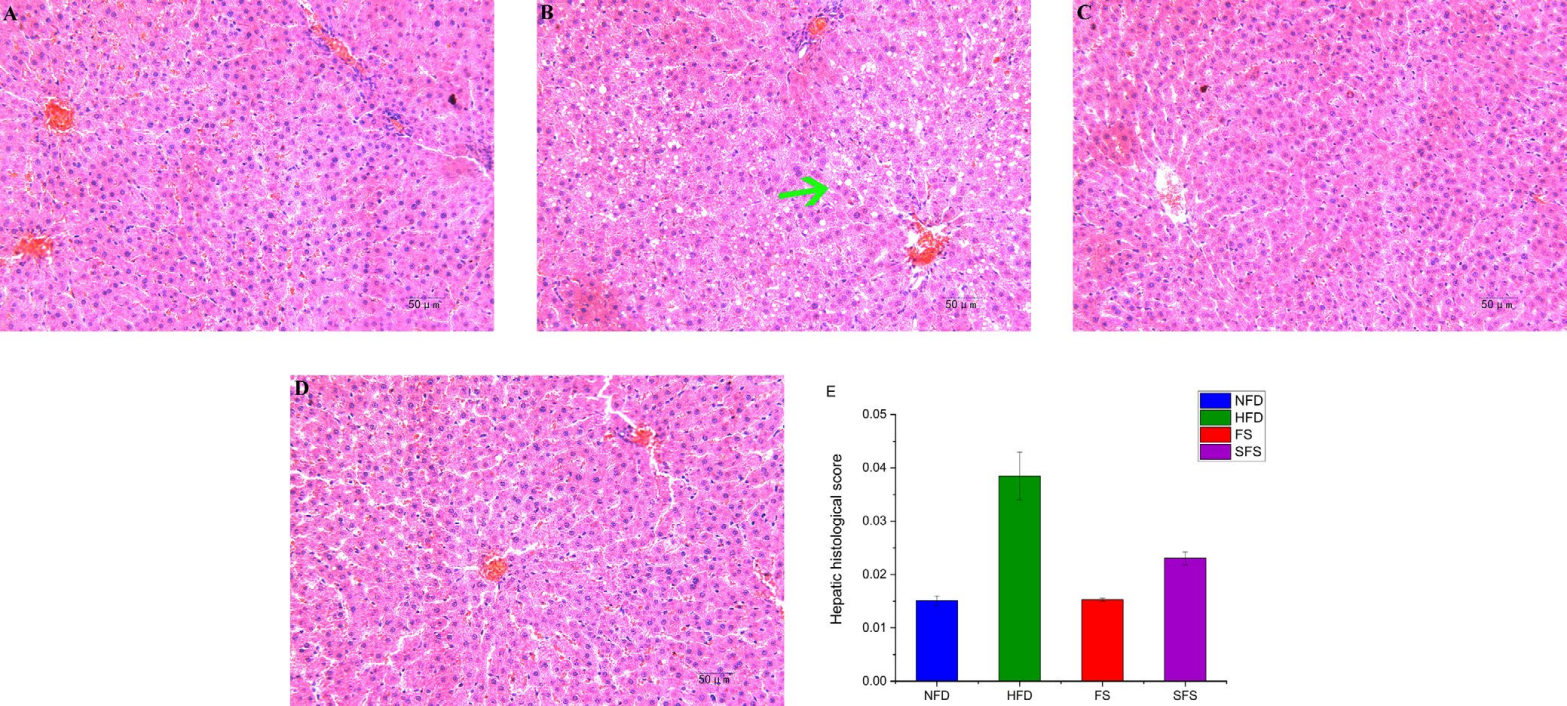
Histopathological analysis of liver tissue of rats in (A) NFD, (B) HFD, (C) FS, and (D) SFS groups at 200× magnification. The green arrow indicated lipid droplet vacuoles in the hepatocytes. (E) Hepatic histological score.

Figure 7 displays the histological morphology of liver sections from each group. In the NFD group, rat liver cells were well‐organized with normal size, clear morphology, and complete cell membrane. Conversely, the HFD group showed a significant number of lipid droplet vacuoles in the hepatocytes, with a large intercellular space and disordered arrangement. However, treatment with FS and SFS significantly improved the morphology of the liver cells, reduced the number of lipid droplet vacuoles in liver tissues, and narrowed the intercellular space. Figure 7E displayed the liver histological score were 60.25% and 40.06% in rats fed with FS or SFS diet, respectively, which were lower as compared to that of the HFD group. This restoration of liver structure suggests that FS and SFS might have potential therapeutic effects on hepatic damage by improved liver lipid accumulation.

### 3.5 Effects of FS and SFS on Intestinal Microbiota

**FIGURE 8:**
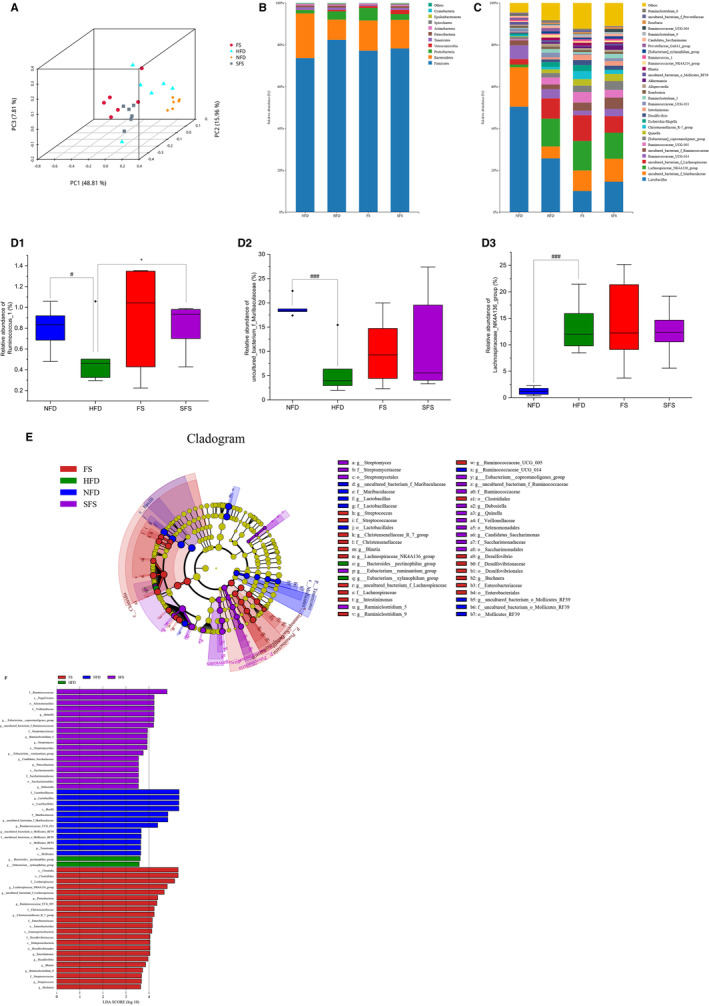
Effect of FS and SFS on the regulates microbiota composition in HFD‐fed rats. (A) Principal coordinates analysis (PCoA) at the genus level. (B) Composition of intestinal microbiota at the phylum level. (C) Composition of intestinal microbiota at the genus level. The relative abundance of (D1) Ruminococcus_1, (D2) uncultured_bacterium_f_Muribaculaceae, and (D3) Lachnospiraceae_NK4A136_group. Based on metastats analysis, significantly different from NFD group, ^#^ *p* < 0.05, ^###^ *p* < 0.001. Significantly different from HFD group, *p < 0.05. (E) LEfSe comparison of gut microbiota among four experimental groups. (F) Linear discriminant analysis (LDA) of four experimental groups.

As shown in Figure 8A, the results of principal coordinate analysis (PCoA) showed that HFD changed the construction of intestinal microbiota. FS and SFS restored the effect of HFD on PC3, and samples from FS and SFS clustered better than HFD, which meant that FS and SFS partially restored the intestinal flora disturbance caused by HFD.

As shown in Figure 8B, the HFD‐diet significantly increased the percentage of Firmicutes (73.65% vs. 82.39%) and Proteobacteria (1.58% vs. 3.97%) but decreased that of Bacteroidetes (21.38% vs. 9.67%) and Actinobacteria (0.66% vs. 0.48%). FS and SFS treatment decreased the percentage of Firmicutes to 77.19% and 78.23%, respectively. They also elevated the percentage of Bacteroidetes to 14.41% and 13.71%, and Actinobacteria to 0.62% and 0.54%, respectively. The effects of FS and SFS on the regulation of Proteobacteria were opposite; FS increased the percentage of it to 6.02%, which was decreased by SFS to 2.73%. In addition, the abundance of Verrucomicrobia was higher in the SFS group (0.70% vs. 2.03%) and lower in the FS group (0.70% vs. 0.86%) as compared with HFD. The ratio of Firmicutes and Bacteroidetes (F/B) of HFD group was higher than that of NFD group (3.44 vs. 8.52). After FS and SFS supplementation, the F/B ratio decreased to 5.36 and 5.71, respectively. This suggests that FS and SFS restored the intestinal microbiota balance at the phylum level.

The relative abundance of microbiota at the genus level was shown in Figure 8C. Levels of genera significantly different between NFD and HFD groups were further analyzed by the Metastats test shown in Figure 8D1 ~ 8D3. Lachnospiraceae_NK4A136_group showed higher abundance in the HFD group, but Ruminococcus_1 and uncultured_bacterium_f_Muribaculaceae showed lower, which indicated intestinal microbial dysbiosis occurred in HFD rats. SFS intervention significantly restored the abundance of Ruminococcus_1, and FS had no significant effect on these genera. Further LEfse analysis and the linear discriminant analysis (LDA) filtered out biomarkers with statistically significant differences (Figure 8E,F), with two genus (Ruminococcaceae_UCG_014 and Lactobacillus) enriched in NFD group, one genus of [Eubacterium]_xylanophilum_group in HFD group, six genus (such as Christensenellaceae_R_7_group et al.) in FS group, and the three genus (such as [Eubacterium]_coprostanoligenes_group et al.) in SFS group.

### 3.6 Correlation Analysis Between Dyslipidemia‐Related Indicators and Intestinal Microbiota

**FIGURE 9:**
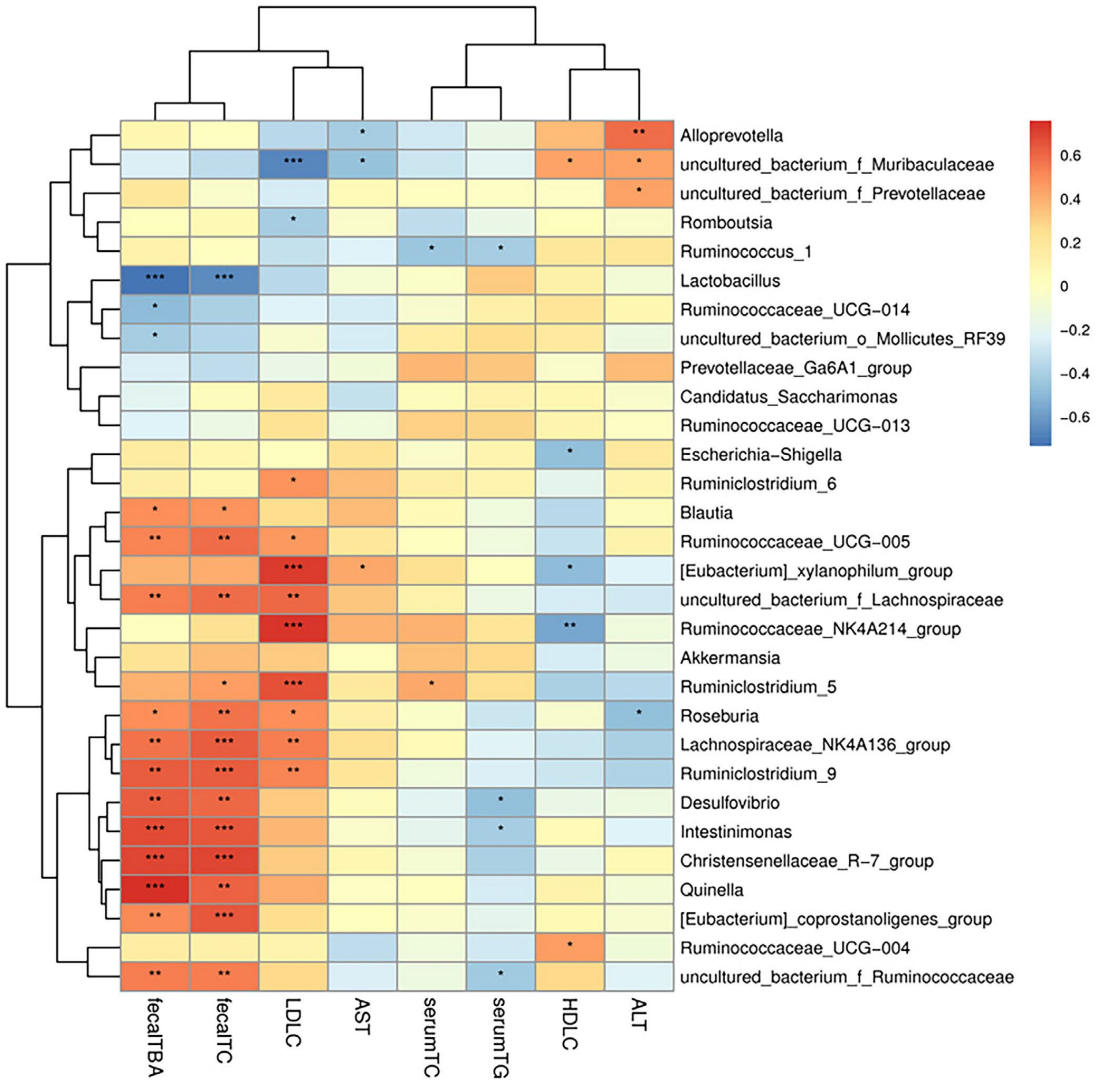
Correlation between dyslipidemia related indicators and intestinal microbiota at the genus level. The intensity of the color represents the degree of association between intestinal microbiota and lipid metabolic parameters. *p < 0.05, **p < 0.01, ***p < 0.001.

In order to compare the effect of FS and SFS on hyperlipidemia, the correlation between lipid metabolism indices and intestinal flora at the genus level was analyed based on Spearman's correlation analysis. As shown in Figure 9, [Eubacterium]_xylanophilum_group which significantly increased in the HFD group, was positively correlated with serum LDL‐C, AST, and negatively correlated with serum HDL‐C. Furthermore, the level of fecal TC and TBA positively correlated with the relative abundance of Intestinimonas, Quinella, Ruminiclostridium_9, Christensenellaceae_R‐7_group, Lachnospiraceae_NK4A136_group, Desulfovibrio, Ruminococcaceae_UCG‐005, uncultured_bacterium_f_Ruminococcaceae, [Eubacterium]_coprostanoligenes_group, and Blautia, while negatively correlated with Lactobacillus, uncultured_bacterium_o_Mollicutes_RF39, and Ruminococcaceae_UCG‐014. In addition, serum TC and TG were negatively correlated with uncultured_bacterium_f_Ruminococcaceae, Desulfovibrio, Ruminococcus_1, and Intestinimonas. Serum HDL‐C was positively correlated with Ruminococcaceae_UCG‐004 and uncultured_bacterium_f_Muribaculaceae, while it was negatively correlated with [Eubacterium]_xylanophilum_group and Escherichia‐Shigella. Therefore, our results suggested that these microbiota were important factors in the regulation of lipid metabolism disorders.

## 4 Discussion

To date, a great number of studies have been performed on the chemical and bioactive investigation of FS. And SFS is the more commonly used form than FS in traditional Chinese medicine (TCM) clinical practice. Studies have demonstrated that processed excipients, such as salt, wine, vinegar, and others, can enhance the dissolution of active compounds in medicinal materials with complex textures, particularly under heat exposure (Liu et al. 2010; Zhan et al. 2011). The qualitative analysis of FS and SFS in the present study demonstrated that the contents as well as the structure of some chemical components changed after salt processing (Wang, Liu, et al. 2022). In this study, the TIC profiles of FS and SFS (Figure 1) show a significant difference in both peak numbers and response values, which might also lead to the different efficacy of them. The tentatively identified components mainly included trigonelline, flavonoids, and steroidal saponins. Since steroidal saponins underwent structural conversion during salt processing and there lacked a quantitative analysis method to determine the contents of total steroidal saponins of different structural characters, only the quantitative determination of trigonelline and flavonoids were carried on. Results of quantatvie analysis indicated that salt processing increased the content of trigonelline. The content of total polysaccharides also increased. It suggested that salt processing facilitated the dissolution of these components from the seed. Furthermore, salt processing enhanced the in vivo absorption of chemical constituents in TCM (Zhang, Zhang, et al. 2022), which helped to explain the differential treatment effects of FS and SFS on hyperlipidemia.

Hyperlipidemia is clinically characterized by abnormally increased blood lipid levels such as TC, TG, or lipoprotein such as LDL‐C. In addition to these commonly used serum markers of hyperlipidemia, the ratio of HDL‐C/TC is also an important indicator of blood lipid metabolism. In recent years, rat models have been widely used to study hyperlipidemia, lipid metabolism, and other metabolic diseases due to their short modeling period, low cost, ease of feeding and management, and high resistance (Qi et al. 2021). Thus, this study used HFD rat models to investigate the therapeutic effects of FS and SFS. There was not a significant difference between these indicators of FS and SFS groups, and factor analysis revealed that on restoring the severity of hyperlipidemia (factor 1), the effects of SFS were comparable to those of FS, suggesting that both of them could improve dyslipidemia.

A meta‐analysis has demonstrated that fenugreek consumption can effectively lower TC, TG, and LDL levels, while increased HDL levels in human blood (Heshmat‐Ghahdarijani et al. 2020), which is consistent with our research findings. Network pharmacology has revealed that various flavonoids in FS were involved in the treatment of hyperglycemia and hyperlipidemia by targeting glucose and lipid metabolism (Banerjee et al. 2019). Flavonoids including schaftoside, isoschaftoside, and vicenin‐1, isolated from FSs, have demonstrated potent lipase inhibitory activity (Fernando et al. 2019). Moreover, isoorientin isolated from fenugreek has shown to reduce lipid accumulation in 3 T3‐L1 adipocyte differentiation, and improve glycolipid metabolism by suppressing adipokines (Luan et al. 2018). These pieces of evidence highlight the importance of flavonoids in the lipid‐lowering activity of fenugreek. In our study, FS and SFS exhibited similar lipid‐lowering effects, which could be attributed to the role of flavonoids in them.

In rats, the cholesterol is converted into bile acids primarily in the liver, with unabsorbed bile acids eliminated in feces. This pathway has a significant impact on the total cholesterol content in rats, and certain drugs can enhance this conversion and excretion process, leading to decreased cholesterol levels (Ning et al. 2020). In our study, the administration of FS and SFS to HFD rats resulted in an increased excretion of TBA and TC in feces, suggesting a potential mechanism for the lipid‐lowering effects of them. Furthermore, factor analysis indicated that SFS was more effective than FS in regulating cholesterol excretion. In our study, the content of polysaccharides in FS increased over 50% after salt processing. Fenugreek gum, composed mainly of galactomannan, is the primary constituent of fenugreek polysaccharides. Studies have shown that galactomannan increased the viscosity of the small intestine, led to inhibition of cholesterol and bile salt absorption (Hamden et al. 2010; Rideout et al. 2008). Additionally, galactomannan has been found to promote the excretion of bile acids and neutral sterols, effectively reduced cholesterol levels (Oh et al. 2022). Fenugreek polysaccharides and oligosaccharides have also been shown to increase fecal TC and TBA levels in hyperlipidemic rats (Srichamroen et al. 2008; Wang et al. 2023). Therefore, the enhancement of bile acid excretion by SFS might be attributed to the increased dissolution of polysaccharides.

In previous studies, it has been shown that FS can regulate oxidative enzymes and improve lipid peroxidation in the liver (Sun et al. 2021; Tewari et al. 2020). In our study, although the liver index of rats in the HFD group did not show significant changes, the level of AST in their serum increased significantly. However, after administration of FS and SFS, the level of AST in the serum and the liver index of HFD rats significantly reduced. Based on factor analysis, AST, as a variable included in factor 1, illustrated the severity of hyperlipidemia. The effects of SFS were comparable to those of FS. This indicated that both FS and SFS had a certain protective effect on liver damage caused by hyperlipidemia. Furthermore, histopathological analysis also revealed the hepatoprotective effects of FS and SFS from liver injury induced by HFD. Many chemical components in FS have been reported to have hepatoprotective activity, such as trigonelline, diosgenin, polysaccharides, 4‐hydroxyisoleucine, and fenugreek oil (Avalos‐Soriano et al. 2016; Fang et al. 2019; Feki et al. 2019; Manasa and Tumaney 2022; Zhang et al. 2015). Due to the complex composition of liver‐protective active ingredients in FS, the mechanism of liver protection from hyperlipidemia of FS and SFS is worthy of further study.

SCFAs are primarily produced through the fermentation of polysaccharides by gut microbiota, although earlier studies have shown that galactomannan and its partially hydrolyzed product increased propionic acid concentrations and reduced acetic acid and butyric acid levels (Shtriker et al. 2018; Tannock and Liu 2020). However, in this study, SCFA levels did not change significantly after administering FS and SFS, likely due to the high molecular weight of the polysaccharides in FS and SFS, which could not be utilized by intestinal microbiota (Mao et al. 2021). BCFAs, which are markers of protein hydrolysis and fermentation by intestinal microbes, are produced from branched‐chain amino acids like valine and leucine, indicated higher protein intake and increased protein fermentation in the large intestine (Macfarlane and Macfarlane 2012; Winther et al. 2021). In this study, HFD‐fed rats had increased the percentage of isobutyric acid and isovaleric acid in cecal content, and administration of FS and SFS resulted in further increase, suggesting that both FS and SFS enhanced protein metabolism.

The intestinal microbiota, which comprises various microorganisms in the intestine, plays a crucial role in the development of hyperlipidemia. Studies have demonstrated that transplanted intestinal microbiota from obese or lean humans induced the same phenotype in germ‐free mice, suggesting that the intestinal microbiota was a potential target for the development of drugs or nutritional interventions for the treatment of hyperlipidemia (Ridaura et al. 2013).

Firmicutes, Bacteroidetes, Proteobacteria, and Actinobacteria are the major bacterial phyla in the intestinal flora, with Firmicutes and Bacteroidetes being the most abundant. Changes in the ratio of their abundances (F/B) can cause highly efficient absorption of calories and subsequent weight gain, even lipid metabolism disorders (Ge et al. 2020; Zheng et al. 2019). Consistent with this, the F/B ratio of HFD rats was significantly higher than that of NFD rats in this study. Administration of FS and SFS led to a decrease in the F/B ratio and an increase in the levels of probiotics from phyla such as Actinobacteria, Proteobacteria, and Verrucomicrobia, which have been proven to be associated with improved gut health (Rodrigues et al. 2022; Xie et al. 2021; Zhu et al. 2022).

At the genus level, HFD‐fed caused changes in the relative abundance of certain genus in the intestine, such as Ruminococcus_1 and uncultured_bacterium_f_Muribaculaceae. Ruminococcus_1 accelerated the conversion of cholesterol to bile acids in HFD mice and thus played a role in regulating cholesterol metabolism (Huang et al. 2019). While uncultured_bacterium_f_Muribaculaceae was negatively correlated with blood lipid levels and improved hepatic steatosis in fatty liver (Al‐Bulish et al. 2022; Hou et al. 2022; Mu et al. 2020). In addition, Ruminococcus_1 and uncultured_bacterium_f_Muribaculaceae could not only cause lipolysis and fatty acid oxidation, inhibit liver cholesterol synthesis, and alleviate host obesity, but also improve insulin sensitivity (Zhao et al. 2021). The results of correlation analysis showed that Ruminococcus_1 and uncultured_bacterium_f_Muribaculaceae were negatively correlated with blood lipid levels, and HFD significantly decreased the abundance of them, but SFS significantly increased the abundance of Ruminococcus_1. Furthermore, although insignificantly, both FS and SFS increased the abundance of uncultured_bacterium_f_Muribaculaceae to an extent. Lachnospiraceae_NK4A136_group is a characteristic genus of gut flora disorders, with higher levels in animals with type 2 diabetes, inflammatory bowel disease, or non‐alcoholic fatty liver disease (NAFLD) (Cui et al. 2019; Wang, Li, et al. 2022; Yao et al. 2022; Zheng et al. 2018). Decreasing the abundance of Lachnospiraceae_NK4A136_group can improve insulin sensitivity and lipid metabolism in HFD rats (Wang, Li, et al. 2022). Similarly, Lachnospiraceae_NK4A136_group in the intestine showed a higher abundance in the HFD group and was positively correlated with serum LDL‐C in this study. But FS and SFS had no effect on regulating the level of it. This suggested that FS and SFS exerted lipid‐lowering effects by recovering HFD‐induced changes in some genus, but they couldn't fully reverse these changes caused by HFD. The LEfSe analysis showed that some probiotics were enriched in the FS and SFS groups. Christensenellaceae_R_7_group, Desulfovibrio, Intestinimonas, Blautia, Ruminiclostridium_9, and Ruminococcaceae_UCG_005 had anti‐obesity, lipid‐lowering effects (Cai et al. 2020; Chen et al. 2018, 2019; Hong et al. 2021; Wang, Zhu, et al. 2022; Zhao et al. 2017). In this study, Christensenellaceae_R_7_group, Desulfovibrio, Intestinimonas, Blautia, Ruminiclostridium_9, and Ruminococcaceae_UCG_005 were enriched in the FS group and showed a positive correlation with fecal TC and TBA levels. [Eubacterium]_coprostanoligenes_group not only scavenged cholesterol by converting cholesterol to coprostanol but also produced sphingosine to regulate host lipid homeostasis (Vlacil et al. 2020; Wei et al. 2021). Uncultured_bacterium_f_Ruminococcaceae belonged to the family Ruminococcaceae. High abundance of Ruminococcaceae led to the reduction of liver cholesterol and plasma TC (Liu, Zhao, et al. 2021). Furthermore, the abundance of the Ruminococcaceae was positively related with bile acid excretion in feces (Liu, Song, et al. 2021). In this study, [Eubacterium]_coprostanoligenes_group, Quinella, and uncultured_bacterium_f_Ruminococcaceae were enriched in the SFS group and showed positive correlations with fecal TC and TBA levels. In conclusion, our study illustrated that the lipid‐lowering effects of FS and SFS were associated with the restoration of Ruminococcus_1 and uncultured_bacterium_f_Muribaculaceae that were decreased by HFD, and their effect on promoting cholesterol and bile acid excretion was related to Christensenellaceae_R_7_group, Desulfovibrio, [Eubacterium]_coprostanoligenes_ group, and other probiotics enriched in FS and SFS groups.

## 5 Conclusions

Salt processing increased the dissolution of polysaccharides and trigonelline from FS. FS and SFS had good therapeutic effects on HFD‐induced hyperlipidemia. Both of them could regulate blood lipid levels, improve the liver damage associated with hyperlipidemia, and promote cholesterol and bile acid excretion. FS and SFS increased the abundance of beneficial bacteria and maintained the homeostasis of intestinal flora. In addition, SFS was more effective than FS in enhancing cholesterol excretion. This provides a reference for the better utilization of SFS as well as FS as alternative drugs for the treatment of hyperlipidemia.

## Author Contributions

**Yang Qu:** conceptualization (lead), formal analysis (lead), writing – review and editing (lead). **Yi Wang:** data curation (lead), investigation (lead), visualization (lead), writing – original draft (lead). **Honghe Xiao:** methodology (equal). **Mingyue Jiang:** data curation (equal). **Qian Cai:** funding acquisition (lead), resources (lead), supervision (lead). **Yi Liu:** validation (equal). **Yu Zheng:** project administration (lead). **Baojie Zhang:** validation (equal).

## Ethics Statement

In this study, all animal experiments complied with the ARRIVE guidelines and were carried out in accordance with the National Research Council's Guide for the Care and Use of Laboratory Animals. Ethical approval for the involvement of animals in this study was granted by the Liaoning University of Traditional Chinese Medicine Research Ethics Committee, Reference number 210000420210205.

## Consent

All authors are aware of and agree to submit the article to this journal.

## Conflicts of Interest

The authors declare no conflicts of interest.

## Supporting information


**Table S1.** Nutritional composition of rats diet.
**Table S2.** Operated parameters of mass spectrometry.
**Table S3.** KMO and Bartlett's test of factor analysis.
**Table S4.** Total variance explained of factor analysis.

## Data Availability

The data that support the findings of this study are available from the corresponding author on request.
