# Supplementary material for: The Chemical Constituents and Anti‐Hyperlipidemia Effect of Salt‐Processed Fenugreek Seed
Source: Food Sci Nutr. 2025 Feb 19;13(2):e70043. doi: 10.1002/fsn3.70043 (PMC11837036; doi:10.1002/fsn3.70043)
Supplement: Supplementary file 1 — Table S1. Nutritional composition of rats diet. Table S2. Operated parameters of mass spectrometry. Table S3. KMO and Bartlett's test of factor analysis. Table S4. Total variance explained of factor analysis. [file FSN3-13-e70043-s001.docx]

**Supplementary Materials**

Table S1. Nutritional composition of rats diet.

|  | HFD | fat emulsion |
| --- | --- | --- |
| Basic-diet | 50.8% | / |
| Water | / | 65.5% |
| Lard | 22% | 10% |
| Egg yolk | 15% | / |
| Sucrose | 10% | 5% |
| Fructose | / | 5% |
| Cholesterol | 2% | 2.5% |
| Pig bile salt | 0.2% | / |
| Glycerol | / | 6.5% |
| Tween 80 | / | 5.5% |

Table S2. Operated parameters of mass spectrometry.

| Parameter | Value |
| --- | --- |
| capillary potential | 3.5 kV |
| fragmentor potential | 75.0 V |
| cone potential | 65.0 V |
| nebulizer pressure | 35 psi |
| desolvation gas flow | 8 L/min |
| desolvation temperature | 350 °C |
| scan mass range | 100~2,000 Da |

Table S3. KMO and Bartlett's test of factor analysis

| KMO and Bartlett's test | | |
| --- | --- | --- |
| KMO Sampling Suitability Quantity | | 0.771 |
| Bartlett's sphericity test | approximate chi-square | 84.881 |
|  | degrees of freedom | 21 |
|  | salience | 0.000 |

Table S4. Total variance explained of factor analysis

| Total variance explained | | | | | | | |
| --- | --- | --- | --- | --- | --- | --- | --- |
| Factor | initial eigenvalues | | | Extract the load sum of squares | | | |
|  | total | Variance percentage | accumulation % | total | Variance percentage | accumulation % | |
| 1 | 3.578 | 51.118 | 51.118 | 3.578 | 51.118 | 51.118 | |
| 2 | 1.786 | 25.507 | 76.625 | 1.786 | 25.507 | 76.625 | |
| 3 | 0.539 | 7.705 | 84.330 |  |  |  | |
| 4 | 0.404 | 5.767 | 90.097 |  |  |  | |
| 5 | 0.348 | 4.978 | 95.075 |  |  |  | |
| 6 | 0.206 | 2.947 | 98.021 |  |  |  | |
| 7 | 0.139 | 1.979 | 100.000 |  |  |  | |
| Extraction method: principal component analysis. | | | | | | |  |
